# Encyclopedia of Infectious Diseases: Modern Methodologies

**DOI:** 10.3201/eid1402.071411

**Published:** 2008-02

**Authors:** Stephen M. Ostroff

**Affiliations:** * Pennsylvania Department of Health, Harrisburg, Pennsylvania, USA

Michel Tibayrenc is someone with big ideas. For many years, he has championed a vision of multidisciplinary systems to approach infectious diseases and public health. One of his longstanding ideas has been to develop a global network of regional institutions built on the model of the US Centers for Disease Control and Prevention. To address routine and emerging disease challenges, this network would blend state-of-the-art molecular approaches such as evolutionary genetics, proteomics, sequencing, and subtyping with traditional field investigations and surveillance.

In support of this vision, Dr. Tibayrenc has edited a book entitled Encyclopedia of Infectious Diseases: Modern Methodologies. The dictionary defines an encyclopedia as “a work that contains information on all branches of knowledge or treats comprehensively a particular branch of knowledge, in articles usually arranged alphabetically by subject” (*1*). When asked to review the book, I was therefore curious as to how he would cover such a broad topic.

Despite its lengthy 747 pages, this book is not an encyclopedia of infectious diseases. First, the content has no obvious pattern, alphabetical or otherwise. As an example, the opening chapter is “Pulmonary Tuberculosis and *Mycobacterium tuberculosis*: Modern Molecular Epidemiology and Perspectives.” Four chapters later, a somewhat redundant chapter called “Molecular or Immunological Tools for Efficient Control of Tuberculosis” appears. In between are unrelated chapters on livestock diseases, HIV/AIDS molecular epidemiology, and uncultured pathogens; these are followed by chapters on leishmaniasis and epidemics of plant diseases.

Second, the book is hardly comprehensive or consistent. It contains full chapters on leishmaniasis, severe acute respiratory syndrome, cholera, hantavirus infection, and Chagas disease, and 2 chapters each on tuberculosis and malaria. Some of these chapters are relatively straightforward reviews; others use the disease for illustrative purposes only. The chapter on livestock diseases has 18 references; the one on leishmaniasis, 402. An important pathogen like *Staphylococcus aureus* is virtually unmentioned; *Streptococcus pneumoniae* does not even appear in the index.

So if the book isn’t an encyclopedia, what is it? The best description would be an interesting potpourri of essays on various aspects of infectious diseases. One chapter is even entitled, “Topical Debates.” Although the emphasis is on pathogen differentiation and evolution, the content runs the gamut from mathematical modeling to geographic information systems to remote sensing to morphometrics. The book even contains a fascinating chapter devoted to archeological epidemiology (mummies) and a whopping 61-page chapter on infectious diseases and the arts, including an extensive list of movies with infectious disease themes.

This assessment by no means trivializes the book. Many of its chapters are extremely well written and do a wonderful job of distilling complex concepts into narrative that even a novice infectious disease scholar could understand. Particularly fine examples are the chapters on influenza evolution and on geographic information systems.

So who would benefit from this book? Not those engaged in clinical medicine and those looking for a practical encyclopedia of infectious diseases; they will be disappointed. This book is fundamentally a loving and personal testament to Dr. Tibayrenc’s vision of multisystems approaches to emerging diseases. Those who share this vision will find a great deal to value in this text.
